# Summer and Autumn Activity of *Eptesicus nilssonii* and Other Bat Species in a High-Altitude Ice Cave in the Tatra Mountains (Southern Poland)

**DOI:** 10.3390/ani16030448

**Published:** 2026-02-01

**Authors:** Krzysztof Piksa, Tomasz Brzuskowski

**Affiliations:** Institute of Biology and Earth Sciences, University of the National Education Commission, Podchorążych 2, 30-084 Kraków, Poland

**Keywords:** Chiroptera, swarming, ice caves, mating behavior, Carpathians

## Abstract

The aim of this study was to examine the role of a high-elevation ice cave (Lodowa Cave in Ciemniak, Mt. Ciemniak, 1715 m a.s.l., Tatra Mountains, Poland) in the seasonal and nightly activity of bats. Bat activity was monitored using mist-netting and direct observations during the summer and autumn seasons of 2008–2011 and 2021. A total of 723 bats representing 10 of the 28 species known from Poland were captured. *Eptesicus nilssonii* predominated (57.5% of the total) and exhibited consistently high activity throughout the study period. Males predominated in almost all species, with *E. nilssonii* showing a strong male bias (85%), while adults were dominant and juveniles were rare (only 1.2% in *E. nilssonii*). Species composition and abundance varied seasonally, and swarming activity occurred between June and November, with a clear peak in July and August. Activity patterns were species-specific: *E. nilssonii* showed a pronounced peak approximately two hours after sunset, while *M. mystacinus* and *P. auritus* displayed distinct temporal activity profiles. A copulating pair of *E. nilssonii* was recorded in September 2011. Our findings suggest that high-altitude ice caves serve as key summer and autumn swarming and mating sites for *E. nilssonii*, highlighting their ecological importance in a warming climate.

## Simple Summary

Bats play a vital role in ecosystems and can serve as early indicators of environmental change. However, their use of high-altitude habitats during the active season remains poorly understood. In this study, we investigated the summer and autumn activity of the northern bat (*Eptesicus nilssonii*) and other species at Lodowa Cave in the Tatra Mountains, southern Poland. To identify the species and study their presence, bats were captured using mist nets near the cave entrance. During the study, 723 bats representing 10 species were captured between June and November. The northern bat was the most abundant throughout the entire season. Several other species, including *Myotis* and *Plecotus*, also regularly used the site for swarming. Activity peaked in mid-summer, and species showed different nightly activity patterns. Our results demonstrate that high-altitude ice caves are crucial swarming and mating sites for cold-adapted *E. nilssonii*. These findings highlight the importance of protecting such unique mountain habitats in a warming climate.

## 1. Introduction

Many bat species are considered subtroglophiles, i.e., organisms that temporarily inhabit subterranean habitats [1]. Caves are used by bats as wintering shelters, night roosts, transitional night quarters, places for forming reproductive colonies, and swarming sites [2,3,4,5]. In the temperate zone of Europe, caves are primarily used as hibernacula from late autumn until spring. Some species use caves as transitional night quarters [4,6] or as night roosts between foraging bouts [7]. In Europe, caves in temperate regions are rarely used as sites for forming reproductive colonies [8]. During summer and early autumn, large numbers of many species of bats gather in the vicinity of cave entrances and within caves themselves. During this period, bats pursue each other, and several different types of activity are observed, such as in-flights and out-flights throughout the cave openings, social vocalization, and mating behavior [5,9,10,11,12,13,14]. This seasonal phenomenon, first described in the 1960s in North America, has been termed “swarming” [15].

Many aspects of this behavior have been investigated, particularly those associated with swarming. However, little is known about bat presence and activity in ice caves or about the ecological role these sites fulfill outside the hibernation period.

The aims of this study were therefore to (1) determine whether bat activity occurs in ice caves between summer and autumn; (2) characterize this activity in terms of species composition, sex ratio, and age structure; (3) analyze temporal patterns of activity both across the season and within the night; and (4) elucidate the potential functional significance of ice caves during this period.

## 2. Materials and Methods

### 2.1. Study Site

Lodowa Cave in Ciemniak (Jaskinia Lodowa w Ciemniaku), located in the Western Tatra Mountains in southern Poland (49°14′ N, 19°53′ W), was selected as the study site. The Tatra Mountains, spanning southern Poland and northern Slovakia, represent the highest range of the Carpathian massif—the highest between the Alps and the Caucasus and between Scandinavia and the Balkan Peninsula. Although the Tatras (highest peak: Gerlach, 2663 m a.s.l.) are considerably lower than the Alps, they exhibit a distinct alpine character and landscape [16].

The cave entrance is situated on the northwest slope of Mt. Ciemniak at an altitude of 1715 m above the timberline, which lies at approximately 1500 m. It is situated near Litworowa Pass (2037 m) and Mułowa Pass (2067 m). The total known length of the cave passages is 390 m with 42 m of vertical extension (−11, +31) [17]. Lodowa Cave in Ciemniak is a typical ice cave in which ice persists throughout the year due to specific microclimatic conditions and is defined by the presence of long-lasting subterranean ice. It is the largest ice cave in both the Tatra Mountains and Poland and hosts an extensive mass of subterranean ice, the volume of which is estimated to considerably exceed 1500 m^3^ [18]. However, the ice inside the cave has exhibited a general decreasing trend since the 1920s, a process that has markedly accelerated in recent decades [19,20].

The air temperature inside the cave remains below 0 °C for most of the year. The rocky slope surrounding and overlying the cave is covered by high-mountain meadow vegetation interspersed with patches of mountain pine (*Pinus mugo*). The mean annual temperature in this altitudinal zone ranges from 0 to 2 °C, and the mean annual precipitation exceeds 1800 mm [21].

### 2.2. Bat Surveys

Field research was conducted between June and October during the years 2008–2011 and in 2021. To minimize disturbance to bats, visits were generally limited to one per month (in 2008, 2010, 2011, and 2021). In 2009 and 2010, however, the cave was visited twice per month. Bats were captured using a single mist net (6 m × 3 m; Ecotone, Poland), consistently placed in front of the cave entrance. The net corridor was only partially enclosed to minimize disturbance to the bats. Mist-netting started before dusk and typically continued until dawn. Captured bats were identified to species and sex, and marked with different non-toxic alcohol-based color marks. They were also aged as adults or juveniles (born that summer) by an examination of the epiphyseal joints in the finger bones [22,23]. The time of capture and the direction of flight (into or out of the cave) were also recorded. The net was monitored continuously, and bats were released almost immediately after capture. All activities were carried out under permits issued by the Polish Ministry of Environment and the Tatra National Park.

### 2.3. Statistical Analysis

Sex ratio trends, as well as the seasonal and nightly dynamics of bat captures, were analyzed using generalized additive models GAMs [24] with smoothing splines, and they are presented with 95% confidence intervals. Differences in the sex ratio were tested with a G-test, which was performed only when the number of individuals exceeded 10 [25].

Species richness and sample completeness were assessed by individual-based rarefaction with 1000 bootstrap iterations [26]. Rarefaction analyses and a G-test were performed in PAST v. 5.3 [27], while GAM fitting was conducted in R v. 4.3.2 using the mgcv package (v. 1.9-1) [28,29].

To avoid pseudoreplication, statistical analyses were based on first captures only; within-night recaptures were excluded. Between-night recaptures were not considered due to long intervals between sessions and non-permanent marks.

## 3. Results

A total of 723 bats, representing 10 of Poland’s 28 bat species, were captured (Table 1). The most abundant species was *Eptesicus nilssonii* (*n* = 416; 57.5%), followed by *Myotis mystacinus* (*n* = 143; 19.8%) and *Plecotus auritus* (*n* = 89; 12.3%). The remaining seven species were captured infrequently (each <5% of total captures).

**Figure 1:**
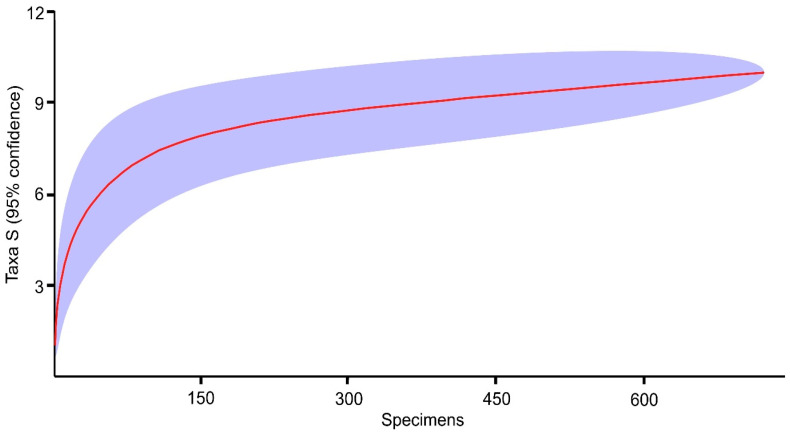
Individual-based rarefaction curve with 95% confidence intervals for bat species richness captured at the entrance of Lodowa Cave in Ciemniak. The red line indicates the rarefaction curve, and the shaded area represents the 95% confidence interval.

The individual-based rarefaction curve rose steeply at low sample sizes and approached an asymptote at 10 species. Approximately 95% of the estimated total species richness was reached with 540–545 individuals, indicating sufficient sampling effort (Figure 1).

**Figure 2:**
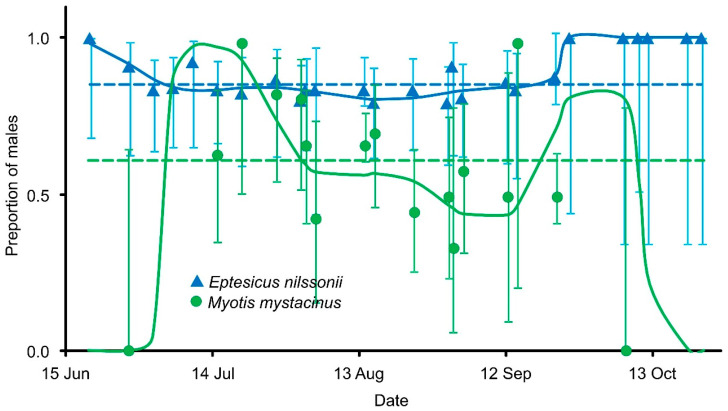
Seasonal dynamics of the sex ratio (proportion of males) in *E. nilssonii* and *M. mystacinus* at the entrance of Lodowa Cave in Ciemniak, based on data pooled across all study years. Points and solid curves (GAM) are shown with 95% confidence intervals; the horizontal dashed line indicates the mean sex ratio.

In all species, males predominated (Table 1). Significant differences in the sex ratio were observed among species; species with small sample sizes and represented by a single sex were excluded from the analysis. For *M. bechsteinii*, *M. nattereri*, and *M. alcathoe*, only males were captured. Seasonal variation in the sex ratio could be assessed for *E. nilssonii* and *M. mystacinus* only. *E. nilssonii* remained consistently male-biased throughout the study period, whereas *M. mystacinus* exhibited seasonal changes in the proportion of males (Figure 2).

**Figure 3:**
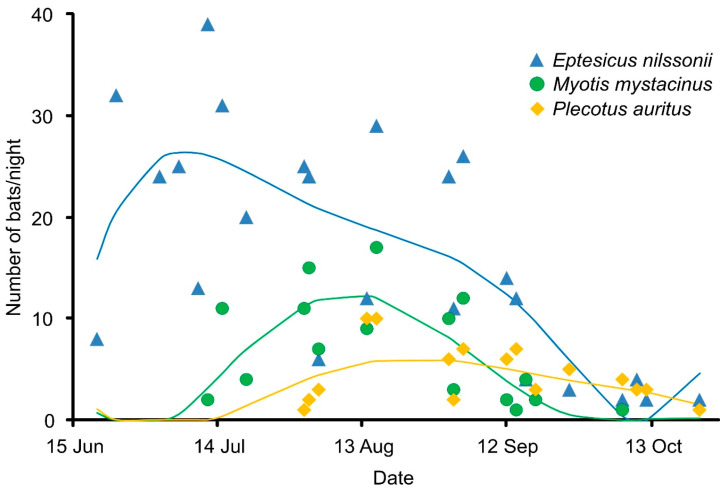
Seasonal dynamics of the three most abundant bat species (*E. nilssonii*, *M. mystacinus*, and *P. auritus*) captured at the entrance of Lodowa Cave in Ciemniak during summer and autumn. Points represent observations, and solid lines represent GAM fits for each species (blue—*E. nilssonii*, green—*M. mystacinus*, yellow—*P. auritus*).

The flight activity of bats at the cave entrance was recorded from mid-June to October, with species-specific timing of peaks (Figure 3). Throughout the study period, the most abundant species was *E. nilssonii*; its activity started in mid-June, peaked in July, and remained high into early autumn. *M. mystacinus* appeared later, from early July, reaching maximum activity in mid-August. *P. auritus* emerged later, in August, reaching its peak around mid-August, followed by a gradual decline towards autumn.

**Figure 4:**
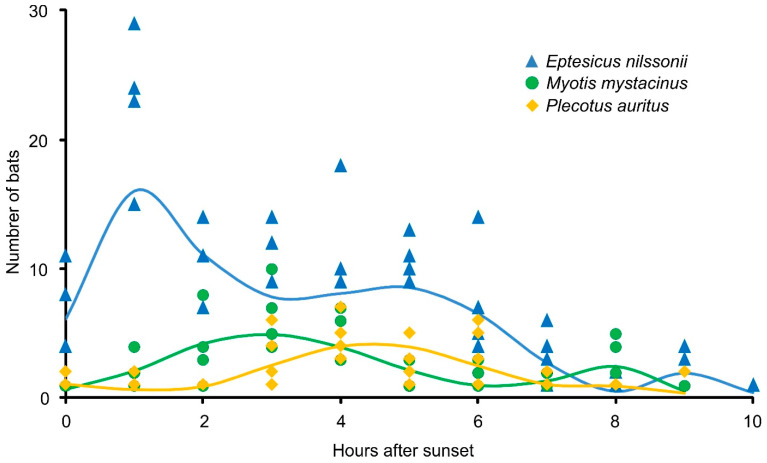
Night-time dynamics of the three most abundant bat species (*E. nilssonii*, *M. mystacinus*, and *P. auritus*) captured at the entrance of Lodowa Cave in Ciemniak during summer and autumn. Points represent observations, and solid lines represent GAM fits for each species (blue—*E. nilssonii*, green—*M. mystacinus*, yellow—*P. auritus*).

The number of bats changed markedly throughout the night (Figure 4). Both *E. nilssonii* and *M. mystacinus* exhibited a bimodal pattern of activity, but the timing of peaks differed between species. In *E. nilssonii*, the first peak occurred shortly after sunset, followed by a second, smaller increase in the middle of the night. In this species, almost 30% of individuals captured during the first three hours after sunset were caught while emerging from the cave, whereas in the other species—except *M. daubentoniid*—this proportion was negligible. In *M. mystacinus*, both activity peaks appeared later—around 2–4 h after sunset and again before dawn. In contrast, *P. auritus* displayed a unimodal pattern, with peak activity between 3 and 6 h after sunset.

Within-night recaptures (i.e., individuals recaptured during the same night) constituted a notable share of capture events. The highest proportion occurred in *E. nilssonii* (21.7%), followed by *M. daubentonii* (20.7%) and *P. auritus* (11.2%). In the remaining species, including the abundant *M. mystacinus*, recaptures were rare, reaching only 4.3%.

Adults predominated in all species. In the most abundant species, *E. nilssonii*, only five juveniles were recorded (1.2%). The highest proportion of juveniles was found in *M. mystacinus* (26.9%). In the remaining species, juveniles were captured infrequently or were absent. Mating behavior was documented in *E. nilssonii* (e.g., a pair was observed in copula on the night of 13 September 2011).

## 4. Discussion

Our results indicate that high-elevation ice caves can serve as both daytime roosts and swarming sites for bats. To date, these caves have primarily been known as hibernation sites for bats, particularly for cryophilous species. Some of these caves, e.g., the Dobšinská Ice Cave [30] and the Demänovské Caves [31], are among the most important hibernation sites for *E. nilssonii* and *M. mystacinus* in Central Europe.

The bat activity observed at the entrance of Lodowa Cave in Ciemniak can be interpreted as swarming for the following reasons: (1) the timing, which was late summer to autumn, when bats swarm in high-elevation caves in the Tatra Mountains [14,32] and other European mountain ranges [33]; (2) a much richer species composition and higher numbers than recorded in this cave in winter; (3) a pronounced male bias [32,34]; (4) very high flight activity in the immediate vicinity of the cave entrance; and, above all, (5) the direct observation of mating behavior.

The most distinctive feature of the swarming bat assemblage at the entrance of Lodowa Cave in Ciemniak—setting it apart from other swarming sites in Poland and elsewhere in Europe—is the marked predominance of a cryophilous, boreal species, the northern bat, *E. nilssonii*. This species has not been recorded during this activity in most regions of Europe, or it has been captured or acoustically detected only in low numbers [5,32,33,35,36,37,38,39,40]. The second most numerous species captured at the entrance to Lodowa Cave in Ciemniak was *M. mystacinus sensu stricto*. Its high abundance is not surprising: across many Tatra caves during swarming, this species is clearly dominant [32], with its proportion reaching up to 88% in one cave [14]. The proportions of the remaining species were broadly consistent with those reported from other Tatra swarming sites [32].

Among our faunal records, the occurrence of *M. alcathoe* is noteworthy. At present, the Lodowa Cave in Ciemniak represents the highest known site for this species within the Carpathians and Central Europe, and is among the highest in Europe. The only record from a higher elevation comes from France, where the species was reported at 2000 m a.s.l. [41].

### 4.1. Seasonal Activity

Swarming at the entrance to Lodowa Cave in Ciemniak starts early, with both the peak and the end occurring earlier than at most European sites. Across most of Europe, swarming typically starts in August, peaks in late August–September, and is minimal or absent in July [4,5,37,42,43]. The early onset of swarming in Lodowa Cave in Ciemniak and the rapid peak of this activity are likely determined by two factors: the cave’s altitudinal location and the high abundance of *E. nilssonii.* Lodowa Cave in Ciemniak is situated within the altitudinal climatic zone classified as “very cool.” The most favorable weather conditions occur in July and August, while from September onward, they gradually deteriorate. The number of days with frost and strong winds increases, and the average night temperature decreases [21]. Consequently, bats inhabiting this area initiate swarming earlier than in lower-altitude sites to take advantage of the brief period of optimal environmental conditions and to complete pre-hibernation activities before the onset of adverse weather. The very high level of bat activity observed near the entrance of Lodowa Cave in Ciemniak as early as July results from the abundant occurrence of northern bats. *E. nilssonii* belongs to a group of bat species that appear the earliest at cave entrances and reach their peak activity sooner than other species [32,38]. A similar advancement of swarming activity, determined by comparable environmental factors, has also been documented in caves located at higher altitudes in the mountain regions of Europe and North America [14,44].

### 4.2. Night Activity

Night-time activity in Lodowa Cave in Ciemniak varied among species. Both *E. nilssonii* and *M. mystacinus* showed a two-peak pattern of activity, although their timing and intensity differed. In *E. nilssonii*, the early peak occurred shortly after sunset and was followed by a secondary increase later in the night. The higher level of activity of this species in the first part of the night likely results from the overlap between movements related to emergence from the cave after diurnal roosting and the onset of swarming behavior. In *M. mystacinus*, both peaks occurred later, suggesting that individuals joined swarming after foraging in nearby habitats. Comparable two-peak patterns of activity—an early peak associated with emergence and a later one linked to swarming and the temporary use of underground sites—have been reported at cave entrances and swarming sites elsewhere, e.g., [36,43,45] as well as in other caves of the Polish Carpathians [32].

In contrast, *P. auritus* exhibited a single, distinct peak of activity between 3 and 6 h after sunset. A similar unimodal pattern for this species has been reported by Furmankiewicz [6], likely reflecting a shorter and more temporally restricted use of the cave for swarming compared with *E. nilssonii* and *M. mystacinus*.

The relatively high proportion of within-night recaptures recorded in *E. nilssonii* supports the idea that some individuals utilize Lodowa Cave in Ciemniak not only for swarming but also as a temporary daytime roost. This view is consistent with the species’ known ecological flexibility and its frequent use of underground shelters outside the strict pre-hibernation period [46,47]. In contrast, other species recorded in the cave—particularly *M. mystacinus* and *P. auritus*—appear to use it mainly for swarming, with only occasional daytime occupancy.

### 4.3. Sex Ratio

One of the characteristic features of swarming—and an almost indispensable aspect of this behavior—is the male-biased sex ratio [5,12,37]. A similar pattern was observed in our study. In almost all bat species, males were considerably more numerous than females, and in some species, no females were recorded at all. Only in the case of *M. mystacinus* was the prevalence of males not as evident as in the remaining species. Interestingly, in this species, a reverse pattern with females prevailing has been reported in the Beskid Wyspowy Mountains [32] and has also been observed temporarily in some caves of the Tatra Mountains [14].

The results of the present study indicate that, during the summer and autumn seasons, even high-altitude ice caves can serve as daytime roosts for bats (primarily for *E. nilssonii*) as well as sites for swarming.

Observations from the studied cave further demonstrate that swarming is a complex phenomenon resulting from multiple interacting processes and serving multiple functions [13,14,48,49]. Its primary role in this type of cave remains difficult to determine. The presence of a copulating pair of *E. nilssonii* suggests that, in this species, swarming activity is associated with mating behavior. However, other functions cannot be excluded. The very low proportion of juveniles in *E. nilssonii* indicates that swarming in this species is unlikely to serve as a mechanism for transmitting information about suitable hibernation or swarming sites. In contrast, the relatively higher proportion of juveniles recorded in *M. mystacinus* may suggest that swarming in this species also facilitates information transfer concerning the location of potential hibernation and swarming sites.

The results presented here suggest that ice caves are key sites for the swarming of *E. nilssonii* in mountain regions. In the caves of the Polish Carpathians, including those located at lower and higher elevations where no permanent ice is present and the microclimatic conditions are milder, this species has either not been recorded at all [32] or has been captured only occasionally [14]. For example, in ice-free caves with a slightly milder microclimate situated close to Lodowa Cave in Ciemniak, such as Czarna Cave 1294 m a.s.l. [32] and Wielka Litworowa Cave 1907 m a.s.l. [14], located at respective distances of 2 km and 1.7 km, *E. nilssonii* was rarely recorded. In the former cave, this species accounted for only 76 individuals out of a total 1859 bats captured [32], whereas in the latter, only nine individuals were recorded among 5608 bats captured [14].

In recent years, within the temperate climatic zone, certain bat species have expanded their distribution ranges northwards and to higher elevations [50]. Such changes have also been observed in the Polish Tatra Mountains, where during the winter seasons, typically thermophilous species such as *Rhinolophus hipposideros* and *M. emarginatus* have been recorded for the first time—species that had not been observed there previously, either in recent decades or during the Holocene. The causes of these changes are generally attributed to ongoing climate warming [51]. One of the consequences of this climatic trend is the progressive disappearance of ice in ice caves across different regions of the world [20,52,53,54]. In the longer term, the substantial reduction in ice volume within ice caves—or its complete loss—together with the resulting alterations in the cave microclimate may lead to significant changes in the composition and structure of bat assemblages using these caves for wintering and swarming. Therefore, the results of the present study may serve as valuable baseline data for future assessments of such changes.

## 5. Conclusions

This study shows that ice caves, despite their harsh conditions, appear to be key swarming sites for cold-adapted *E. nilssonii*. Such environments may become increasingly important for bat assemblages in the context of ongoing climate change.

## Figures and Tables

**Table 1:** Number of bats of each species caught in the Lodowa Cave in Ciemniak. Number of surveys is given in parentheses; sex ratio (proportion of males in total) is provided in brackets. Used symbols: *—male-to-female sex ratio significantly different from unity (*p* ≤ 0.05); “-” indicates absence of the species. Only bat species with abundance ≥ 10 were included in the analysis. In total, 6 *E. nilssonii*, 2 *M. mystacinus*, and 1 *P. auritus* were sex-undetermined (escaped before sex determination).

Species	2008 (4)	2009 (8)	2010 (6)	2011 (7)	2021 (5)	Total
*M. myotis*	4[0.75]	2[1.0]	2[0.0]	3[1.0]	1[1.0]	12[0.75]
*M. bechsteinii*	-	-	-	1[1.0]	-	1[1.00]
*M. nattereri*	2[1.0]	1[1.0]	1[1.0]	1[1.0]	4[1.0]	9[1.0]
*M. mystacinus*	28[0.61]	35[0.71] *	23[0.48]	21[0.71] *	36[0.59]	143[0.62] *
*M. brandtii*	3[1.0]	3[1.0]	4[0.75]	2[1.0]	5[1.0]	17[0.94] *
*M. alcathoe*	-	1 [0.0]	-	-	-	1[0.0]
*M. daubentonii*	6[0.67]	8[0.75]	3[0.67]	6[0.83]	6[0.83]	29[0.76] *
*E. nilssonii*	77[0.83] *	113[0.84] *	72[0.85] *	94[0.87] *	60[0.87] *	416[0.85] *
*V. murinus*	2[1.00]	1[1.0]	1[1.0]	2[1.0]	3[0.67]	9[0.78]
*P. auritus*	13[0.77]	32[0.87] *	8[0.75]	20[0.90] *	16[0.94] *	89[0.86] *
Total	135	196	114	150	128	723

## Data Availability

The data presented in this study are available in the Supplementary Materials of this article.

## Supplementary Materials

The following supporting information can be downloaded at: https://www.mdpi.com/article/10.3390/ani16030448/s1, Table S1. Dataset used for the analyses of bat activity and sex ratio.
